# Chemical and Light Inducible Epigenome Editing

**DOI:** 10.3390/ijms21030998

**Published:** 2020-02-03

**Authors:** Weiye Zhao, Yufan Wang, Fu-Sen Liang

**Affiliations:** Department of Chemistry, Case Western Reserve University 2080 Adelbert Road, Cleveland, OH 44106, USA; wxz394@case.edu (W.Z.); yxw1413@case.edu (Y.W.)

**Keywords:** epigenome, epigenome modifications, chemically induced proximity, light control

## Abstract

The epigenome defines the unique gene expression patterns and resulting cellular behaviors in different cell types. Epigenome dysregulation has been directly linked to various human diseases. Epigenome editing enabling genome locus-specific targeting of epigenome modifiers to directly alter specific local epigenome modifications offers a revolutionary tool for mechanistic studies in epigenome regulation as well as the development of novel epigenome therapies. Inducible and reversible epigenome editing provides unique temporal control critical for understanding the dynamics and kinetics of epigenome regulation. This review summarizes the progress in the development of spatiotemporal-specific tools using small molecules or light as inducers to achieve the conditional control of epigenome editing and their applications in epigenetic research.

## 1. Introduction

Chemical modification patterns on histone tails and DNA collectively constitute the epigenome that dictates unique gene expression patterns and resulting phenotypes in each distinct cell type [1,2,3]. The alterations of the epigenome due to the dysregulation of epigenome pathways and mutations of epigenome regulators contributes to pathogenesis of various human diseases including many developmental diseases and cancers [4,5,6,7,8,9,10,11,12,13,14,15,16,17,18,19]. Several epigenetic regulatory mechanisms including DNA methylations, histone tail posttranslational modifications (PTMs), ATP-dependent chromatin remodeling and non-coding RNAs have been shown to play key roles in governing gene activities [6,7,15,20,21,22,23,24,25,26,27,28,29]. 

Genome-wide mapping of epigenome modifications by the next generation sequencing technologies has provided valuable information correlating specific epigenome modifications or combinations with certain transcriptional activities [25,30]. However, to unequivocally delineate the function of each epigenome marks and to establish the causal relationship between epigenome modifications and associated gene activities, tools to selectively manipulate these epigenome marks for functional investigation are required. Conventional approaches manipulating the levels or activities of epigenome modifiers, including genetic manipulation (i.e., overexpression or knockout/knockdown) and pharmacological intervention (i.e., using small molecule inhibitors) [31], typically lack gene specificity and lead to global changes in the epigenome making it difficult to determine whether the observed effects are due to local epigenome changes or from secondary effects caused by remote changes. Furthermore, lacking temporal control in locus-specific altering of epigenome marks makes it difficult to interrogate the highly dynamic epigenome regulatory events [32]. In vitro biochemical assays allow well-controlled epigenome environment to be set up for functional studies [33]. However, such isolated and simplified systems are not likely to reconstitute the endogenous cellular environments and all the regulatory pathways involved. To address these limitations, several innovative tools in achieving genome locus and temporal specific epigenome editing in living cells have been developed in recent years. In this review, we will summarize the chemical biology strategies for developing chemical or light inducible and reversible epigenome editing platforms and their applications in controlling gene activities and mechanistic studies of epigenome regulation. 

## 2. Overview of Epigenome Editing

Epigenome editing aims to write or erase specific epigenome modifications at specific gene loci to reprogram local epigenome environments and alter resulting gene expression. Programmable locus-specific DNA-targeting technologies play a pivotal role in realizing this goal [34,35,36], which enable the genome locus-specific recruitment of specific epigenome modification protein domains, proteins or protein complexes (i.e., epigenome modifiers) resulting in the remodeling of local epigenome environment [36,37,38,39].

Several DNA-targeting technical platforms have been developed that have been or can potentially be applied to locus-specific epigenome editing [37,40,41,42,43]. Three most commonly used techniques for DNA targeting in cells include zinc finger proteins (ZFPs), transcription activator-like effector (TALE) and clustered regularly interspaced short palindromic repeats (CRISPR) systems. Zinc finger domains containing conserved Cys2His2 (C2H2) motifs have been engineered to selectively recognize any combination of three DNA base pairs. Combining tandem modules of these ZFP domains each with unique sequence specificity, specific DNA sequences in the genome can be targeted [37,44]. Instead of recognizing DNA triplets, a 34-amino acid TALE domain recognizes one unique base pair of DNA and different TALE module can be combined in specific orders to form an array recognizing specific DNA sequences [37,45]. Unlike ZFPs and TALEs that rely on protein-DNA base recognition, the CRISPR-based system relies on Watson/Crick base-pairing between the programmable single guide RNAs (sgRNAs) and the target DNA, which greatly simplifies the process to implement DNA targeting and allows a broader use of the technology [37,46,47]. For example, the widely used CRISPR/Cas9 system utilizes short synthetic sgRNAs consisted of a customizable 20-nucleotide sequence complementary to the target DNA sequence preceding a conserved 5′ protospacer adjacent motif (PAM) and an RNA scaffold that Cas9 protein binds. While the wild type Cas9 endonuclease induces DNA double strand breaks, the catalytic inactivated mutant (dCas9) can be fused to any effectors including epigenome modifiers, which can then be recruited to specific genome loci via custom sgRNAs and result in localized biological effects [46,47]. 

**Figure 1:**
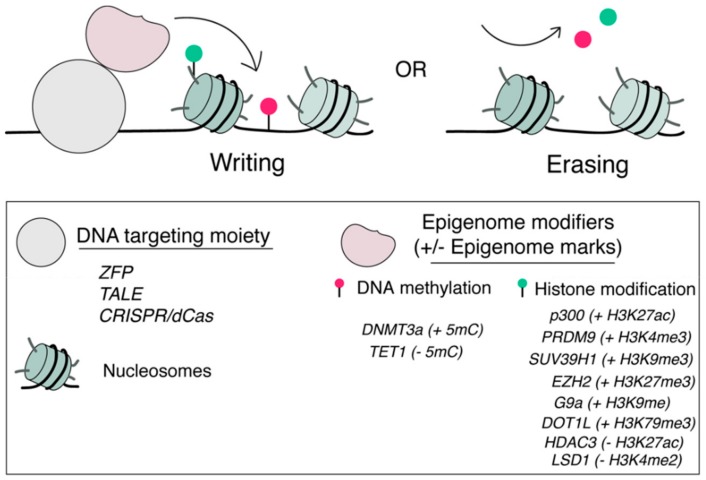
Locus-specific tools for epigenome editing. Epigenome modifiers can be recruited to a pre-defined genomic locus through a locus-specific DNA targeting moiety including ZFPs, TALEs and dCas9s for artificial writing (+) or erasing (−) specific epigenome marks including DNA methylation and histone PTMs.

These strategies have been applied to target histone tail modification proteins to specific genome loci leading to the precise editing of a specific histone tail PTMs (Figure 1). For example, p300 histone acetyltransferase (HAT) core domain [48] that catalyzes acetylation of histone H3 lysine 27 (H3K27ac) was fused to the catalytically inactive dCas9 from *Streptococcus pyogenes*, dCas9 from *Neisseria meningitidis*, TALEs or ZFPs and was recruited to the promoter and/or enhancer region of *IL1RN*, *MYOD*, *OCT4*, *HS2*, *HBE*, *HBG* or *ICAM1* gene leading to the installation of H3K27ac and the activation of corresponding genes in human HEK293T cells [49]. Alternatively, using MS2 aptamer loop-fused sgRNAs, MS2-p300 fusion protein can be recruited to the *IL1RN* locus and induced gene activation [50]. Targeting G9A H3K9 methyltransferase to the promoter of *HER2* in HCT116 cells increased H3K9me3 but did not lead to the transcriptional repression of *HER2* [51]. Further studies of combinational targeting with EZH2 and other epigenome modifiers to *HER2* revealed that long-term epigenetic memory can be achieved but in a context-dependent manner [52]. Several other epigenome modifiers have also been targeted with similar approaches for editing histone tail PTMs, including DOT1L [53], PRDM9 [53], HDAC3 [54], EZH2 [49], SUV39H1 [51], and G9A [51]. 

Similar strategies have also been used to recruit DNA methyltransferase and demethylase activity to defined genome loci to achieve de novo methylation or demethylation of CpG DNA sequences and modulate gene activities [55,56,57,58,59,60,61,62,63]. For example, the lysine-specific demethylase 1 (LSD1) was fused to either TALE [64] or dCas9 from *Neisseria meningitidis* [65] and targeted to putative enhancer loci of *OCT4*, *SCL* or unknown genes in human K562 erythroleukemia cells or mouse embryonic stem cells (mESCs). Localized LSD1 fusion protein resulted in the demethylation of H3K4me2 and modulation of the regulatory activity of individual enhancer elements with high specificity. Using these tools, not only putative candidate enhancers for a gene can be functionally annotated, the unknown target gene of an enhancer can also be revealed [64,65]. It was shown that directed DNA methylation by TALE-DNMT at *CDKN2A* promoter locus decreased its expression and increased replication of primary human fibroblasts [56]. The targeted demethylation of the BDNF promoter by dCas9-TET1 induced BDNF expression in post-mitotic neurons, while targeted demethylation of the *MYOD* distal enhancer activated MyoD expression and facilitated re-programming of fibroblasts into myoblasts [59]. When DNMT3a was recruited to the CTCF zinc finger protein binding sites across the genome, it induced local de novo methylation of CpGs, which interfered CTCF-mediated looping function [59]. 

In addition to directing epigenome modifiers specific for editing particular epigenome marks, transcriptional regulators with broader effects on local epigenome environments have also been recruited to alter gene activities accompanied with changes in multiple epigenome marks. These transcriptional regulators, including activators (e.g., the herpes simplex viral protein 16 (VP16), its oligomers (VP64), p65 domain derived from human NF-kB protein, Rita, VPR) [66,67,68] and repressors (e.g., Krüppel associated box (KRAB) domain) [66,67], have been fused to ZFPs, TALEs and CRISPR/dCas9s to target specific genome loci. Although these regulators do not directly edit specific epigenome marks, they recruit multiple chromatin and epigenome modifying proteins that cause larger scale changes of the local epigenome environment leading to either gene activation or silencing [49,65,66,67,68,69].

Although directly targeting epigenome modification proteins or transcriptional regulators via ZFP, TALE or CRISPR/dCas9 platform addresses the issues of global epigenome changes when using conventional genetic or pharmacological methods, these tools lack the vital temporal controls required to dissect the dynamics and kinetics of epigenome regulations, establish the causal relationship between specific epigenome marks and gene activities, and determine the interplays between different epigenome marks or mechanisms. To tackle these limitations, several inducible and reversible epigenome editing tools controlled by cell permeable small molecules or light have been developed that offer precise temporal controls in addition to spatial controls (i.e., gene locus specificity) as discussed above. 

## 3. Conditional Epigenome Editing

Several chemical biology strategies have been adapted in epigenome editing to achieve ligand-or light-dependent recruitment of epigenome modifiers to specific genome loci. Here, we discuss the available inducible platforms in epigenome editing and their applications in mechanistic and functional studies. 

### 3.1. Chemically Induced Proximity (CIP)-Based Editing

CIP technologies have been integrated in a variety of ways to offer temporal controls in epigenome editing and regulation. In the CIP system, a small-molecule inducer promotes the homo- or hetero-dimerization of two corresponding inducer-binding adapter proteins that are individually fused to two proteins of interest (POIs). By controlling the proximity of POIs, various downstream biological processes can be triggered upon the addition of the inducer [70,71,72,73,74,75,76,77]. Furthermore, many of these CIP systems are readily reversible and the induced dimerization/biological effects can be reversed upon the removal of the inducer from the system [76]. To date, several natural occurring or synthetic small-molecules and their corresponding binding protein pairs have been reported to provide orthogonal CIP systems with distinct properties such as different association/dissociation kinetics and responsive dosages [78,79,80]. By integrating CIP technologies, various epigenome editing effectors including histone tail PTM modifying proteins and chromatin remodeling complexes have been targeted to specific genome loci to reprogram local epigenome environments in temporally controlled fashions. 

**Figure 2:**
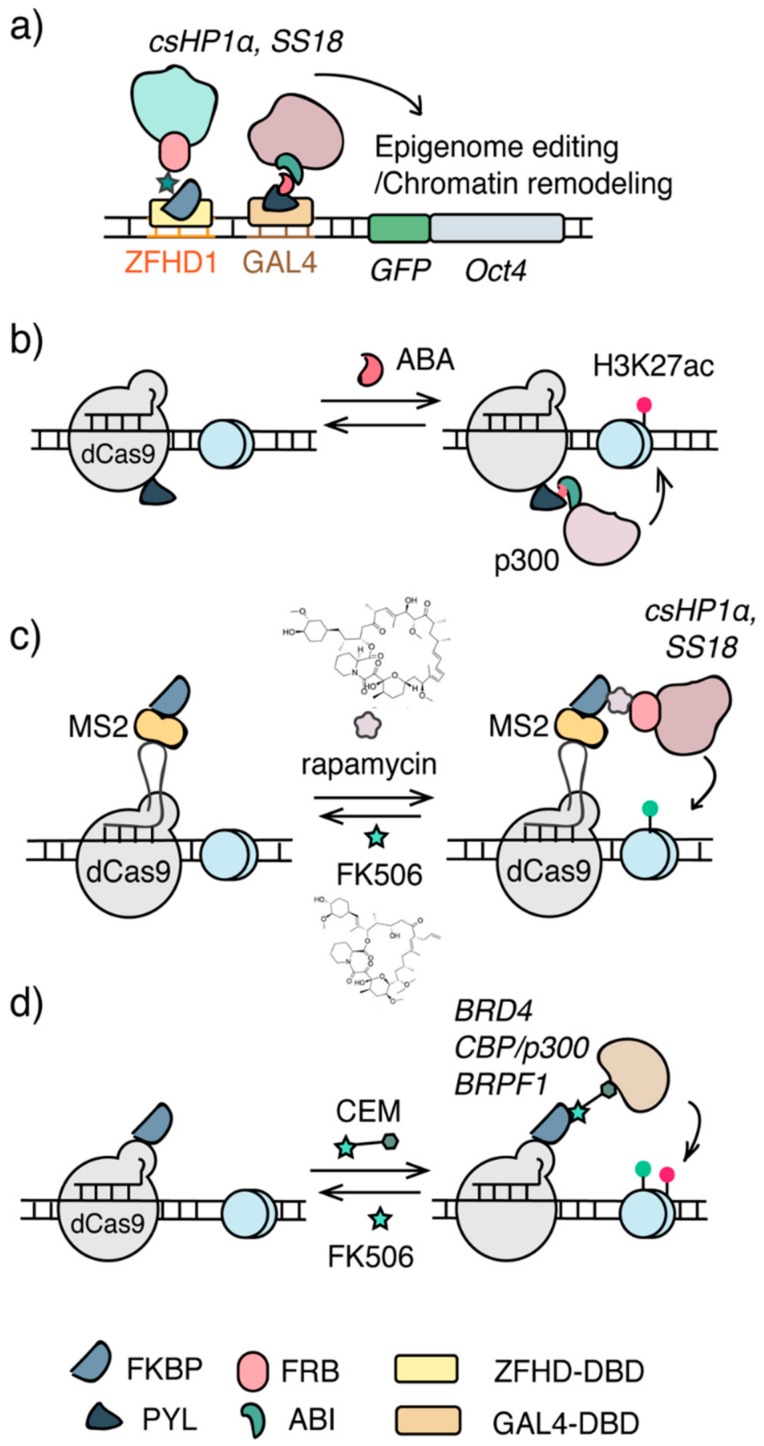
Chemical inducible approaches for epigenome editing. (**a**) The Chromatin in vivo Assay (CiA) system. Arrays of DNA binding sites (e.g., GAL4, ZFHD1) were knocked-in upstream to the promoter of *OCT4*. Epigenome modifiers were recruited via DBD and CIP. (**b**) The CRISPR/dCas9-CIP integrated epigenome editing system. Acetyl transferase p300 core domain was recruited to specific genomic loci to install H3K27ac upon addition of ABA. (**c**) The FIRE-Cas9 system. CRISPR/dCas9 was combined with rapamycin CIP to recruit csHP1α and ss18/BAF for epigenome editing and chromatin remodeling. (**d**) Chemical epigenetic modifier (CEM), a bifunctional molecule containing an FKBP-binding molecule FK506 and a molecule recognizing a specific effector, recruits endogenous epigenome effectors to the targeted genomic locus for epigenome editing.

To study the dynamics of heterochromatin formation and epigenetic memory, the Chromatin in vivo Assay (CiA) system was engineered in mESCs. In this system, two arrays of DNA binding domain (DBD) recognition sequences, including 12 copies of ZFHD1 DBD binding sequences and five copies of GAL4 binding sequences, were inserted upstream to the *OCT4* promoter for targeting purposes. In addition, an in-frame nuclear *EGFP* reporter was knocked on to replace the first exon of *OCT4* at one allele of the OCT4 gene [81]. (Figure 2a) In the CiA system, a CIP inducer, rapamycin, was used to dimerize FKBP12 and FRB fusion proteins individually fused to GAL4-DBD and the chromo-shadow domain of HP1α (csHP1α), which directly interacts with H3K9-specific histone methyltransferases. The rapamycin-dependent recruitment of csHP1α to the CiA:OCT4 locus led to increased H3K9me3 at the targeted locus within 18 h and spread cross a 10 kbp region after 5 days. It was accompanied by increased DNA methylation, decreased H3K4me3 and H3K27ac, and resulted in the formation of heterochromatin and silencing of the *EGFP* reporter. The precise temporal control introduced by the CIP method in csHP1α recruitment enabled kinetic studies to quantify the speed of H3K9me3 spreading and the heterochromatin formation (0.18 nucleosomes/hour) [81]. Furthermore, another CIP system based on abscisic acid (ABA), which dimerizes PYL and ABI fusion proteins, was employed to recruit VP16 at the CiA:OCT4 locus to oppose the repressive marks and re-activate EGFP expression. Combining these two orthogonal CIP systems, the dynamics and the stability of the artificially established H3K9me3 and heterochromatin were studied [81].

To investigate how mSWI/SNF chromatin remodeling complexes and Polycomb repressive complexes (PRCs) oppose each other to maintain the homeostasis as well as the mechanisms in resolving and forming heterochromatin [82], a similar CiA system was used to target the Brg/Brm-associated factor (BAF) complex to the CiA:OCT4 locus in mouse embryonic fibroblasts (MEFs), which is occupied by Polycomb complexes (Figure 2a) [83,84]. To recruit BAF complexes, SS18, a subunit of the BAF complex, was fused to FRB. Upon the addition of rapamycin, SS18-FRB, along with the associated BAF complex, was dimerized with ZFHD1-FKBP12 fusion protein and targeted to the CiA:OCT4 locus in MEFs. The recruitment of BAF complexes resulted in the rapid ATP-dependent eviction of PRC complexes [83]. With the temporal control provided by the rapamycin inducible system, the sequence of epigenome remodeling events triggered by BAF targeting at the locus can be monitored in high temporal resolution, revealing the immediate loss of PRC1 and PRC2 upon BAF occupation, paralleled by decreased H2AK119Ub and followed by the loss of H3K27me3 [83]. It was also discovered that tumor-suppressor and oncogenic mutant BAF complexes have different effects on PRC eviction [83] and disease-associated mutations in Smarca4 (or BRG1, the ATPase of the BAF complex) disrupt the eviction of PRC1 [85]. Furthermore, using the same CIP-controlled BAF complex recruitment system, it was found that topoisomerase II (TOP2) synergized with BAF complexes to resolve as well as re-formation of facultative heterochromatin [84].

The CiA system described above utilized a knock-in DNA sequence at the *OCT4* locus to enable the recruitment of epigenome modifiers through corresponding DBDs, which limits the inducible epigenome editing or remodeling to occur only at this particular locus. To offer a more versatile temporal-controlled epigenome editing platform, CRISPR/dCas9 was combined with the ABA-based CIP method to temporally target the ABI-fused p300 HAT core domain via PYL-fused dCas9 to the promoter region of *IL1RN*, *MYOD1*, *GRM2* and *HBA* genes in human HEK293T cells leading to increased H3K27ac and gene activation (Figure 2b) [86]. With the fast dimerization kinetics that ABA CIP offers, the p300 HAT recruitment, H3K27ac installation and mRNA expression can be monitored following its precise time course, allowing the establishment of their causal relationship [86]. Furthermore, the reversibility of the ABA CIP system allowed the rapid removal of inducer and the dislodge of P300 HAT from the targeted locus, offering an opportunity to assess the stability of artificially installed H3K27ac and a model to study epigenetic memory [87,88]. Similarly, a FIRE-Cas9 system was developed using sgRNA/dCas9 for genome targeting and the rapamycin CIP method to induce the recruitment of epigenome effectors [89]. In this system, sgRNA was fused to MS2 stem loops that were recognized by MS2-FKBP12 fusion proteins. Upon rapamycin addition, SS18-FRB or csHP1α-FRB fusion proteins can be dimerized with MS2-FKBP12 and be recruited to the sgRNA targeting locus (Figure 2c). Induced targeting of csHP1α to the promoter of *CXCR4* in HEK293 cells or to the OCT4 locus in mESCs led to increased H3K9me3 and decreased *CXCR4* and *OCT4* expression, which was reversible with the addition of FK506, a competitive binder to FKBP12 [89]. When recruiting SS18/BAF complexes, it was found that it was sufficient to oppose Polycomb complexes within minutes, leading to the activation of bivalent gene transcription in mESCs [89].

In addition, multiplex inducible methods combining orthogonal CIPs and dCas9s have been applied to target transcriptional modulators to multiple gene loci to control gene activities [90,91], which can be readily adapted for multiplex site-specific epigenome editing by targeting epigenome modifiers. 

### 3.2. Chemical Epigenetic Modifier (CEM)-Based Editing

In CIP-based epigenome editing systems, epigenome modifiers to be recruited have to be fused to a CIP inducer dimerizable protein and be delivered/expressed in cells. It may cause overexpression of these epigenome modifiers that elevate non-specific background editing and impede in vivo application due to increased cargo size when using viral delivery methods. To address these concerns, synthetic bifunctional ligands, termed chemical epigenetic modifiers (CEMs), were developed to target endogenous epigenome modifiers (Figure 2c). The first generation CEMs conjugate FK506 (a ligand binds FKBP12) to histone deacetylase (HDAC) inhibitors that recruits HDAC-containing corepressor complexes [92,93]. Using these CEMs in the CiA system with the GAL4DBD-FKBP12 fusion protein, endogenous HDAC3 can be targeted to the CiA:OCT4 locus leading to the reduction of H3K27ac and gene silencing in revisable and dose-dependent manners [92]. To expand the CEM strategy for broader applications, a second generation CEM platform was recently developed that incorporated the CRISPR/dCas9 system for DNA targeting [93]. In this new approach, instead of fusing FKBP12 to GAL4DBD as in the first generation CEM, FKBP12 was fused to either dCas9 or MS2 protein that can be targeted to endogenous genome loci through standard sgRNAs or MS2 loop-fused sgRNAs. Using these CRISPR-mediated targeting methods, different synthetic CEMs, with FK506 linked to ligands/inhibitors for BRD4, BRF1 or CBP/p300, were shown to induce dose-dependent and reversible activation of multiple genes, including *MYOD1*, *CXCR4*, *IL1RN* and *OCT4*, through recruiting endogenous epigenome modifiers (Figure 2d) [93].

### 3.3. Light Inducible Epigenome Editing

**Figure 3:**
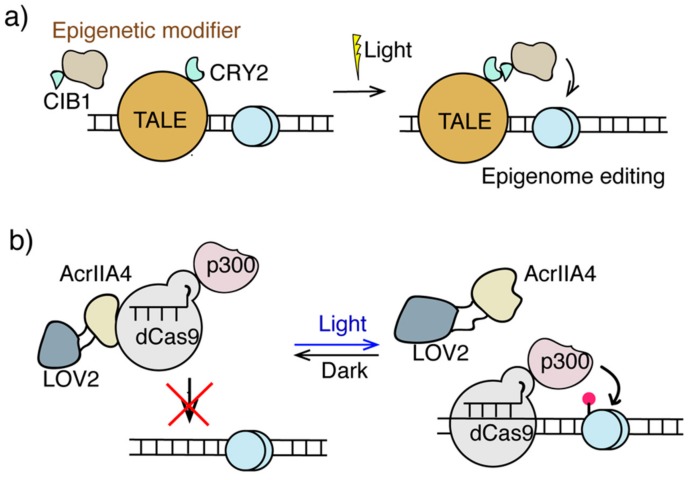
Light inducible epigenome editing. (**a**) The LITE and epiTALE system. Light induces dimerization of CRY2 and CIB1 to direct epigenome modifiers to TALE-targeting sites for epigenome editing. (**b**) The CASANOVA system used light responsive LOV2-AcrIIA4 to target dCas-p300 for H3K27ac editing.

Light inducible transcriptional effectors (LITEs) have been developed to modulate the transcriptional dynamics and local epigenome landscapes of endogenous genes (Figure 3a). In the LITE system, plant-derived light responsive CRY2 and CIB1 proteins dimerize upon photo irradiation at 466 nm. Combining LITE and TALE, epiTALEs were constructed by fusing CRY2 and CIB1 individually to a TALE genome targeting module and to VP64 or a variety of epigenome effectors including HDACs, methyltransferases (HMTs), HAT inhibitors, as well as HDAC- and HMT-recruiting proteins. Upon light irradiation, these epiTALEs altered levels of H3K9me1, H4K20me3, H3K27me3, H3K9Ac, and H4K8Ac, and repressed *GRM2* and *NEUROG2* expression in primary neurons and Neuro2a cells [94]. A similar strategy, termed LACE (Light-activated CRISPR/Cas9 effector), applying CRY2/CIB1 light inducible system but replacing TALE with CRISPR/dCas9 for DNA targeting, was used to recruit VP64 for gene activation and can potentially be adapted to recruit epigenome modifiers for epigenome editing [95].

Another light-controlled platform, termed CASANOVA (CRISPR-Cas9 activity switching via a novel optogenetic variant of AcrIIA4), has been developed comprising the anti-CRISPR protein AcrIIA4 and the photo-sensor LOV2 domain (Figure 3b) [96]. In this method, the LOV2 domain was inserted into AcrIIA4, which in the dark state preserved AcrIIA4 native conformation and therefore its inhibition to Cas9. Once exposed to blue light, LOV2 changed its conformation leading to the disruption of the conformation and Cas9-inhibitory function of AcrIIA4. This light responsive property of LOV2-AcrIIA4 fusion protein allows the CASANOVA system to control the targeting of Cas9 to genomic DNA with light stimulation. CASANOVA was also used for light inducible epigenome editing, which enables light-controlled targeting of dCas9-p300 leading to the activation of the *IL1RN* gene [96].

The CIP method can potentially also be adapted for light inducible epigenome editing. Several light-inducible methods based on CIP have been reported using CIP inducers caged with different photosensitive groups. These caged CIP inducers (including ABA, rapamycin, gibberellic acid, and other synthetic dimerizers) [77,97,98,99,100,101,102,103,104,105,106,107,108] were inactive for dimerization in the caged form but can be uncaged to regenerate active inducer upon light irradiation. This photo-controlled version of CIP should be readily adaptable for light inducible epigenome editing. With the availability of orthogonal CIPs and dCas9s coupled with a variety of photo-caging groups responsive to different light wavelengths to choose from [109], it is possible to develop multiplex light-controlled tools for independently editing at multiple genome loci.

### 3.4. Inducible Regulation of Higher-Order Epigenome Organization

**Figure 4:**
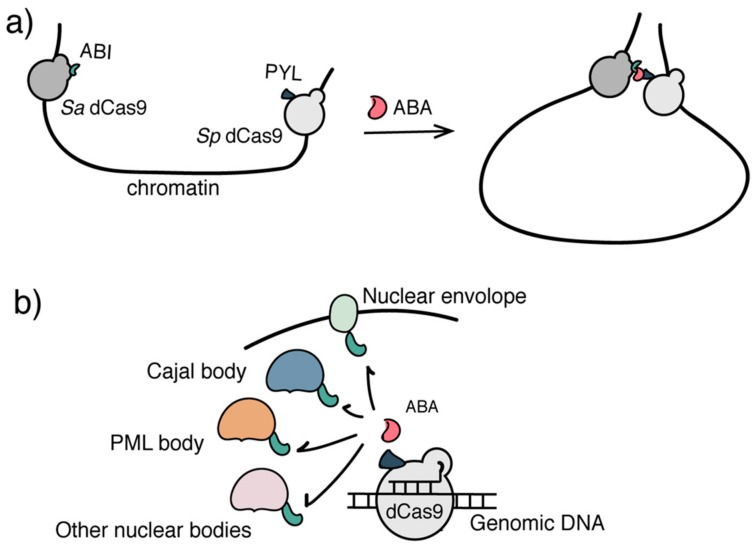
Inducible systems to regulate higher-order epigenome organization. (**a**) The CLOuD9 system used two orthogonal CRISPR/dCas9s to target distal genomic loci. ABA induced the juxtaposition of two loci to organize a particular 3D chromatin architecture. (**b**) The CRISPR-GO system used ABA to induce the sub-nuclear localization of genomic DNA/chromatin of interest.

Regulating the dynamic three-dimensional (3D) organization of chromatin within the cell nucleus is another layer of epigenome regulatory mechanism to control gene activities [19,20,110,111,112,113]. Tools for artificial manipulation of higher-order chromatin structures can facilitate their functional studies. To address this need, a platform termed CLOuD9 combining CRISPR/dCas9 with ABA-based CIP has been developed to control chromatin looping (Figure 4a) [114]. In the CLOuD9 method, two orthogonal dCas9 proteins from *Staphylococcus aureus* (*sa*Cas9) and *Streptococcus pyogenes* (*sp*Cas9) were fused individually to ABA-binding adaptor proteins PYL and ABI and targeted to two distal genomic loci mediated through corresponding sgRNAs. Adding ABA induced the juxtaposition of these two targeted chromosomal loci to form a particular chromatin spatial organization. It was demonstrated that using the CLOuD9 system, adding ABA resulted in the formation of stable but reversible chromatin loops that brought the promoter of β*-globin* into contact with the *HS2* locus control region (LCR), which led to increased β-globin expression in K562 cells [114].

Another inducible system, CRISPR-genome organizer (CRISPR-GO), was developed by combining CRISPR/dCas9 with ABA-based CIP or the Trimetho-prim-Haloligand (TMP-Htag) inducible DHFR/HaloTag system [101], to control the 3D organization of chromatin through sub-nuclear localization [115] (Figure 4b). In the CRISPR-Go system, ABI-fused dCas9 was targeted to various repetitive or non-repetitive genome loci by sgRNAs. Distinct protein marks of various sub-nuclear compartments including nuclear envelope, PML and Cajal bodies were individually fused to PYL, which, upon ABA addition, can induce reversible recruitment of sgRNA/dCas9 targeted chromatin loci to corresponding sub-nuclear locations. It was found that the trans-localization of genomic loci to nuclear periphery and Cajal bodies repressed expression of reporter and endogenous genes [115].

## 4. Other Inducible Approaches Applicable to Epigenome Editing

**Figure 5:**
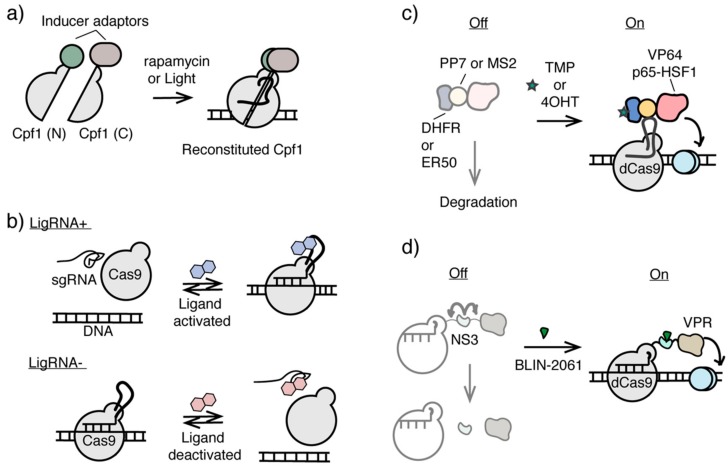
Inducible CRISPR/Cas systems potentially applicable for epigenome editing. (**a**) Inducible split Cpf1 systems controlled by chemical inducer or light. (**b**) Ligand activated or deactivated sgRNAs for conditional CRISPR/Cas9 control. (**c**) Ligand-controlled stability of dCas9 fusion protein using ligand-dependent destabilizing domains. (**d**) The stability of protease NS3 domain integrated dCas9 fusion protein can be controlled by NS3 inhibitor BLIN-2061.

Cas12a, or Cpf1, has been engineered as a complementary genome editing platform along with Cas9 [116,117,118]. A split version of Cpf1 has recently been developed to enable chemical or light-induced reconstitution of active Cpf1 [119]. In this inducible Cpf1 system, the N-terminal and C-terminal Cpf1 fragments were individually fused either to rapamycin dimerizable FRB and FKBP12 proteins or to blue light inducible dimerization domains, termed the Magnet system [120]. With rapamycin addition or 470-nm light irradiation, two split halves of Cpf1 can be brought into close proximity and reconstituted into functional Cpf1 at sgRNA-targeted loci for genome editing [119]. The split version of the catalytic inactivated Cpf1 (dCpf1) has also been developed allowing the inducible and reversible recruitment of transcriptional regulators to control gene activity in vitro and in vivo [119], which should be adaptable to generate both chemical and light inducible epigenome editing systems (Figure 5a).

Another approach of constructing an inducible CRISPR/Cas9 system is to develop ligand-controlled activation and deactivation of sgRNAs, which controls the association between Cas9 and sgRNA. Different RNA aptamers were inserted into the sgRNA sequences and subjected to functional screening to identify either ligand-activated sgRNA (termed ligRNA^+^) or ligand-inactivated sgRNA (termed ligRNA^−^) using theophylline or 3-methylxanthine as the small molecule inducer (Figure 5b) [121]. Although this platform was shown to successfully regulate gene expression in bacteria, it is so far not transferable to eukaryotic systems without further optimization. Nevertheless, it is a promising direction that may be used to create inducible epigenome editing systems with minimal interference at the targeted locus.

In addition to the strategies of using ligands or light to control the recruitment of epigenome modifiers to the targeted genomic loci, modulating the stability of dCas-fused epigenome editing effectors can potentially be another useful tool for conditional epigenome editing. Ligand-dependent destabilizing domains (DDs) have been used to regulate the stability of various POIs in cells [122,123]. Without ligands, unstructured DDs cause DD-fused POIs to undergo proteolytic degradation, whereas ligand-stabilized DDs prevent DD-fused POIs from degradation [122,123]. Using DDs engineered from dihydrofolate reductase (DHFR) and estrogen receptor (ER50), the level of dCas9-fused transcriptional activator including VP64 and P65-HSF1 can be orthogonally controlled by trimethoprim and 4-hydrotomaxifen in dosage-dependent and reversible manners (Figure 5c) [124]. This strategy in conditional control of dCas9-activator stability has been used to induce cellular reprogramming and differentiation [125], and can potentially be applied for ligand inducible epigenome editing.

Another approach to control the level of functional dCas9-effector fusion protein and may be adaptable for conditional epigenome editing is to use ligand-dependent NS3 protease domain derived from hepatitis C virus (HCV). Inserted between dCas9 domain and the effector domain, NS3 promoted the cleavage between these two domains, unless an antiviral NS3 inhibitor BLIN-2061 was present, which inhibited NS3 activity and maintain the full length of the functional dCas9 fusion protein (Figure 5d). Using this system, the activity of dCas9-NS3-VPR can be controlled by BLIN-2061 in dosage-dependent and reversible manners to regulate CXCR4 expression [126].

## 5. Conclusions and Perspective

Advances in epigenome editing technologies have led to a technical revolution facilitating functional epigenomic research and contributing to therapeutic development for diseases caused by epigenome dysregulation. The genome locus-specific targeting enabled by ZFPs, TALEs and CRISPR/dCas offers unparalleled precision in epigenome editing unachievable by conventional genetic or pharmacological approaches. Conditional editing platforms controlled by small molecule ligands or light further open up the door for high resolution temporal manipulation and dissection of epigenome regulatory processes, which enable the quantitative analysis of the kinetics and dynamics of epigenome establishment, remodeling and maintenance. The knowledge gained from these studies and the editing tools developed for these purposes will directly contribute to the discovery and development of novel epigenome therapy strategies [4,13,16,18,127].
